# Round the Hospitals

**Published:** 1920-02-14

**Authors:** 


### ROUND THE HOSPITALS.

Princess Mary is to lay the foundation-stone on May 12 of a new Nurses' Home in the grounds of the Royal Hospital and Home for Incurables at Putney. The Princess has consented to receive purses containing five guineas or upwards towards the cost of the new building, and it is hoped there will be a large concourse of young people at the ceremony of laying the stone eager to lay their offerings in Her Royal Highness's hands The new home will be a great boon to the nursing staff, who need plenty of recreation and fresh ideas after their labour among the incurable cases in the home. An excellent work is carried on here in the training of nurse attendants for aged and chronic patients. It is a branch of work which the ordinary trained nurse seldom takes to very kindly, but there is none which affords more scope for the exercise of a true vocation. It is particularly suited to women

**ROUND THE HOSPITALS**—*(continued)*.

who take up nursing after thirty. The excellent tone and true spirit of humanity which prevails in the Putney Home renders it an admirable training-ground.

We learn that the annual meeting and conference of the College of Nursing will be held on June 17 and 18, 1920, at the Royal Society of Medicine, 1 Wimpole Street, W.

We note with satisfaction that the number of Queen's nurses admitted with the approval of Queen Alexandra to Queen Victoria's Jubilee Institute for Nurses on January 1 amounts to 115. These include some 25 members of the Scottish branch and three of the Irish branch.

The latest news of the *Daily Telegraph* Fund for Nurses is that the interest in it is growing in different parts of the country, and that collective efforts are being made in a large number of places, and particularly among business houses in the City of London. One bank has promised 1,000 shillings if eleven others will do likewise. Many entertainments are being organised for the benefit of the fund in addition to the Grand Carnival to be given at the Holland Park Skating Rink on February 18. The Nurses' Own Day is next Monday, February 16, when the columns will be taken up with acknowledgment of gifts from matrons, sisters and nurses in institutions and private work all over the country. All the big hospitals have set themselves to the task of sending in 1,000 shillings as a token of their hearty approval of the action of Lord Burnham in setting the Fund on foot no less than of the objects it has in view. Sir Harold Boulton, who contributed on February 7 a valuable testimony to nurses' needs and nurses' qualities, brings into special prominence the onus which rests on the public to provide for pre-war nurses, who were expected, he declares, to give the results of a comparatively long and special training in return for a miserable pittance during a comparatively short period.

The Scottish Board of Health has caused severe disappointment in Glasgow by refusing to sanction the proposal of the parish council to extend the nurses' accommodation at Duke Street Hospital. The Council, with Mr. Andrew Stewart as chairman, has decided by eighteen votes to six to protest. The ground for the new nurses' home was acquired some time ago. It is a property known as the London Arms, adjoining the hospital, and the Council had agreed to demolish the existing buildings and erect a new block for the use of the nurses who are in "crying need" of more accommodation at the Duke Street Hospital. There are cases, it was stated, of two and even three nurses occupying the same room, and the danger of this makeshift arrangement is illustrated by the fact that when one nurse caught measles her two room-mates took the infection from her. It can hardly be believed that the facts relating to the lack of accommodation for the nurses were adequately represented to the Scottish Board of Health.

The members of Queen Alexandra's Royal Naval Nursing Service have at length been granted an increase to their pay. It is not entirely satisfactory, for it takes the form of a bonus, whereas permanent additions to the rate of pay were long overdue. Head sisters will receive a bonus of 17s. 6d. a week; superintendent sisters, 12s. 6d., and nursing sisters 8s. 9d. a week. The grants are retrospective from December 1, and *Truth* is asking why this should be the order for the Naval nurses when the Army nursing services received corresponding increments from August 31 last. We do not learn that the hours on duty have been reduced, but this is a matter which affects the well-being of the service fully as much as the rate of pay—perhaps even more.

A correspondent draws our attention to the fact that there is a strong feeling among "The Comrades of the Great War" that temporary disability pensions should be made permanent two years after the original award, and we understand that the Ministry of Pensions, having been approached, has the matter under consideration, though we must not be taken to endorse the suggestion. Our correspondent urges that the Minister of Pensions should be informed that disabled nurses would also welcome permanent pensions after two years of temporary awards. There can be no doubt that nurses are sometimes left out of schemes which might benefit them; witness their exclusion for many months from the benefit of the King's Fund. It is eminently within the scope of the College of Nursing to make representations on this subject to the Ministry of Pensions, and we trust the matter may be taken up by this body, which is entitled to speak for nurses in general. The matter of alternative pensions, in which nurses are at a disadvantage as compared with both officers and men, is one which might also be ventilated.

The conditions of service in the Birkenhead Union Infirmary are to be improved by the institution of a 56-hour week for both night and day staff. The improvement is hailed with much satisfaction in the Press, and was not brought about too soon if the facts related in the *Birkenhead News* be accurate. It is stated that the night staff in this infirmary of 300 beds consisted of thirteen nurses who went on duty at 8.30 and left the wards at 8 a.m., sometimes not till 9 a.m., without any break. After their dinner, if there were operations, it is stated that they went in groups of threes and fours to the theatre, where they finished duty at noon, or possibly not till 2 p.m., according to the number of operations. The writer, "Interested," inquires "Can thirteen overworked nurses do their duty to 300 patients?" If these statements be exaggerated let us hear the truth without delay. As they stand

**ROUND THE HOSPITALS—**(*continued*).

they furnish a powerful argument for the inclusion of all nurses under the Hours of Employment Bill.

---

The typical generosity of nurses towards each other and towards their patients is often dwelt upon by people who send contributions to the Fund. One contributor says that the outstanding characteristic of nurses is their unstinted unselfish charity towards their patients, so that they have been known to deprive themselves of necessities in order to provide luxuries for those they tend. A Scottish nurse urging the need for better pay that nurses may secure their own independence, pertinently adds: "But are we to think only of the future nurse and leave the others to struggle along as best they may?" A doctor who sends his contribution confesses that by right in many cases of recovery the nurse ought to have had his fee. Many sad cases are brought to light of nurses disabled by their past work and reduced to great straits. One district nurse crippled with rheumatism and bedridden lies in a ward of the workhouse infirmary, having no means and no pension. Another, on whose foot a bomb fell during an air-raid on London in which she was giving her services, is only kept from the workhouse by the gift of a few shillings a week from a friend. The varied record of brave patient women reduced to hopeless and helpless poverty long before old age is reached is of a nature to touch all hearts. The eyes of the public are being opened at last to some of what may be termed the normal catastrophes of the nursing profession. As we go to press the amount contributed totals 106,139 shillings.

---

Bradford folk are noted for their generosity, but never have they given a more conspicuous lead to the philanthropic than in their recent decision to offer hospitality to a thousand starving children from Central Europe. The Finance Committee of the Bradford Corporation, meeting on February 5, resolved upon this fine course. The invitation of the City Council is for a period of twelve months. The children are to be of school age and selected from the famine areas. The Education Committee is to be instructed to include the little guests in the schools, and to arrange for their having school meals, clothing, and foot-gear. A careful medical record is to be kept of every child from beginning to end of the visit by the school medical staff. As to the housing difficulty, advertisements are to be inserted in the local papers inviting Bradford citizens to house one or more of the children, and, of course, the success of the scheme will largely depend on the extent to which this voluntary aid is forthcoming. It will open up a new and interesting branch of work to the Bradford nurses.

---

Sisters and nurses should not forget that the post-graduate course of lectures on Tuberculosis begins at the Brompton Hospital for Consumption on February 17 at 8 p.m., and continues every Tuesday and Friday evening during February, March, and April. The opening lecture is on "The History of Tuberculosis," by Dr. Burrell; the lecture on the 20th is by Dr. Inman on "The Cause of Tuberculosis." The fee for the course of nineteen lectures is £1 1s.; that for a single lecture is 2s.

---

The need in America for home helps is pressing, and the scarcity of assistants for the graduate nurses so serious that the Mayor of Chicago has attempted a very daring experiment to meet the demand. A six weeks' course of instruction in nursing has been carried out under Dr. J. D. Robertson, the City Commissioner of Health, who has instituted a "training-school for home and public health nursing." Eight hundred women have come forward to receive instruction, and the Mayor has presented to all who completed the course with credit a certificate to that effect. Something like consternation has seized the training-schools and graduates at this invasion, from which many fear that grave consequences may ensue. It remains to be seen whether the health authorities of the city can keep the 800 helps in their proper department—strictly subordinate to the trained nurses and well adapted for domestic matters. But there is no doubt that these impressive certificates are apt to go to the head and produce a very perceptible swelling.

---

We learn that more volunteers are needed at the Splint and Artificial Limb Depôt, 123 Church Street, Chelsea, S.W. 3. The depôt was started during the war by the Surgical Requisites Society, under the inspiration of two women sculptors, who worked out the various processes. It has now been handed over to the Red Cross, and the work hitherto undertaken solely in the interests of soldiers is to be developed for the benefit also of crippled children and disabled civilians. All kinds of devices for bringing the maimed in hand and foot back to normal activity have been thought out, for instance, a valuable apparatus for gradually bending stiff elbow and wrist joints. Papier-maché splints have been brought to a high pitch of perfection. There is a metal room where fittings are made and a large department for making artificial limbs of compressed wood fibre. It is skilled work but easily learnt, and nursing sisters in receipt of a pension, but anxious still to be of use to the suffering, would be welcomed, and find it full of absorbing interest.

---

The Central Committee on Women's Training and Employment attached to the Ministry of Labour has been made a Standing Committee, charged with the duty of planning and carrying out schemes of work and training for women who are unemployed or whose earning powers have been lessened owing to their services in the war. The residue of the Queen's "Work for Women" Fund will be used to pay the cost of training and the Executive Committee of the National Relief Fund

**ROUND THE HOSPITALS—(*continued*).**

have allocated £500,000 for the same purpose. The Committee will give maintenance scholarships to enable women to become teachers of domestic science or physical culture or undertake public health work, or enter the professions of law and medicine. There is hope of extending this aid to some 5,000 women. The Education Board is short of 9,000 women teachers, and it is in contemplation to give short intensive courses of instruction to women whose education is suitable in order to qualify them for work in the elementary schools.

---

A very pleasant evening was spent on February 2 by upwards of one hundred members of the staff of No. 14 General Hospital, Wimereux, who dined together at the Victory Restaurant, Leicester Square. Lieut.-General Sir John Goodwin, who was formerly commandant of the hospital, presided, and General Sir Arthur Sloggett, late Director of Medical Services in France was also present. Dame Maud McCarthy, G.B.E., was unfortunately unable to attend. The company were greatly pleased by the receipt during the evening of a telegram from H.M. the King as follows: "I am commanded to express the thanks of the King to the staff of No. 14 General Hospital, Wimereux, for their kind message addressed to His Majesty on the occasion of the dinner, which, under the presidency of General Goodwin, their former and distinguished commandant, has re-united that noble band of men and women who ministered to the wounded and sick in one of the largest hospitals in France. The King recalls with pleasure his visit in October 1915, to the Wimereux Hospital, and recognises that the same spirit of devotion and comradeship which animated their work in those days has brought them together again this evening. His Majesty congratulates them on this happy reunion."

---

Miss Charlotte Aikens concludes her adventures among the South American hospitals in the January number of the *Trained Nurse*. She gives us, in her account of Lima, a gratifying glimpse of two English nursing sisters, Miss Soper and an unnamed colleague, who came out at the earnest request of the city authorities to bring order into the Dos de Mayo Hospital for Men, with 700 beds. Prior to their arrival the only attention given to the fifty patients in each ward was from an untrained woman and two working-men. They did the rough work of the wards, and in the same aprons, and often without washing their hands, helped at operations. Miss Soper has got together a class of young men and young women to form the nucleus of a training-school. When she began six towels were provided for each fifty patients, and were allowed to be washed but once a week. The rule was for four beds to get a change of linen each week, with the result that their turn did not come round again for three months. When the sisters asked for unheard-of quantities of soap, and insisted upon hands and faces, at least, being washed once a day, the Peruvians, even the doctors, were deeply shocked at the waste of time and water. The writer may well say: "All the nurse heroes did not go into war work. Two of them can be located in Lima."

---

The Stratford Nursing Home held its annual meeting under cheerful circumstances on January 28. The lady superintendent, Miss Cottam, was released from her post as matron of the Stourbridge Military Hospital last July, and the Home has returned to its pre-war state. The treasurer is able to show a balance of £166, as compared with an almost similar deficit the year before. The Home performs a varied work. It received 209 patients during the year, supported a district nurse for gratuitous work among the sick poor in Stratford-on-Avon, and sends out four fully-trained nurses for private cases. The Home has been managed, both internally as regards the superintendence and externally as regards its finances, with exceptional ability, and the measure of its success is shown by the remarkable enthusiasm of the Stratford people in placing it on a sound basis during a year of great stress and scarcity.

---

The worst has happened. The nurses at the Carrickmacross Fever and General Hospital have struck. The patients lie untended. One, recently admitted, has died. No temporary nurses are forthcoming and the situation is as bad at it can be. The insurgents, for such they must be termed, are demanding a minimum salary of £52 a year with "subsistence allowance" of £70. Whether these women are merely probationers or aspire to be called sisters and staff nurses we have no means of knowing. It will be a public calamity if their barbarity towards the patients they had contracted to tend is rewarded.

---

Progress at the West Bromwich Infirmary is evidenced by the appointment of four additional probationers and one male nurse to the staff in order to provide for the establishment of an eight-hour day for the nurses such as is already enjoyed by the other officials. Two additional cubicles are to be erected at the Lodge for their accommodation.

---

The forty-third annual report of the Leeds Trained Nurses' Institution announces the close of war conditions and the return of many members of the staff. The number of nurses now employed totals fifty-four, and they attended 444 cases in the year. The staff needs considerable augmentation, for no fewer than 370 cases had to be refused. Is it possible that all the returned nursing sisters, whose cause we pleaded last summer, have now procured engagements? Or does the Leeds Institution fail to attract war sisters to its staff? We note that £8,100 has been added to the Pension Trust Fund for the benefit of nurses.

